# Sick of the Sick Role: Narratives of What “Recovery” Means to People With CFS/ME

**DOI:** 10.1177/1049732320969395

**Published:** 2020-11-11

**Authors:** Anna Cheshire, Damien Ridge, Lucy V. Clark, Peter D. White

**Affiliations:** 1 University of Westminster, London, United Kingdom; 2 Queen Mary University of London, London, United Kingdom

**Keywords:** chronic, illness and disease, experiences, illness and disease, qualitative, recovery, adaptation, coping, enduring, qualitative, United Kingdom

## Abstract

Little is known about what recovery means to those with chronic fatigue syndrome/myalgic encephalomyelitis, a poorly understood, disabling chronic health condition. To explore this issue, semi-structured interviews were conducted with patients reporting improvement (*n* = 9) and deterioration (*n* = 10) after a guided self-help intervention, and analyzed via “constant comparison.” The meaning of recovery differed between participants—expectations for improvement and deployment of the sick role (and associated stigma) were key influences. While some saw recovery as complete freedom from symptoms, many defined it as freedom from the “sick role,” with functionality prioritized. Others redefined recovery, reluctant to return to the lifestyle that may have contributed to their illness, or rejected the concept as unhelpful. Recovery is not always about eliminating all symptoms. Rather, it is a nexus between the reality of limited opportunities for full recovery, yet a strong desire to leave the illness behind and regain a sense of “normality.”

## Introduction

Chronic fatigue syndrome/myalgic encephalomyelitis (CFS/ME) is a long-term health condition which involves a complex range of symptoms. A characteristic feature is postexertional malaise or fatigue. This fatigue is described as being different to the kind of tiredness/fatigue experienced by healthy individuals or pre-illness, and includes unpredictable exhaustion not alleviated by rest and an inability to think (Olson et al., 2015). Other symptoms include sleep disturbance, muscle/joint pain, headaches, and cognitive dysfunction (National Institute for Health and Care Excellence [NICE], 2007). The severity of symptoms varies between individuals and over the course of the illness (Anderson et al., 2012). There is no consensus as to whether CFS and ME are the same or different conditions (Brurberg et al., 2014), but we use the combined term CFS/ME, as per the U.K. NICE criteria (NICE, 2007). CFS/ME is relatively common: it is estimated that it affects around 0.76% of the population (Johnston et al., 2013). Impact on individuals often involves a significant loss in physical, social, and occupational functioning. In addition, people may experience skepticism about—and lack of knowledge of—CFS/ME among members of the medical community, family, and friends (Anderson et al., 2012; Bayliss et al., 2014). It is estimated to cost the U.K. economy £102 million a year in lost earnings alone just for those who attend specialist services (Action for ME, 2014; Collin et al., 2011).

Prognosis for CFS/ME is uncertain. For adults, full recovery appears uncommon, with one review reporting an average of 5% of people reaching this status (Cairns & Hotopf, 2005). Prospects for experiencing an improvement in symptoms are better, with studies reporting improvements in 8% to 63% of patients (Cairns & Hotopf, 2005). Undertaking treatments (e.g., cognitive behavior therapy or graded exercise therapy) can improve chances of recovery (Knoop et al., 2007; White et al., 2013). However, recovery is a complex construct, with factors including population studied, methodology, the particular health condition or disability (including definition of CFS/ME), definition of recovery, and patient narratives all influencing interpretations. Defining recovery in the case of CFS/ME is additionally complicated because reports of continued fatigue are not necessarily linked to CFS/ME, and conversely those whose symptoms have temporarily remitted are not necessarily completely recovered (Cairns & Hotopf, 2005). Additionally, definitions of recovery from CFS/ME appear to be predominantly shaped by researchers and professionals rather than patients. Devendorf et al. (2019) found that physicians conceptualized recovery as a complete remission of symptoms and a return to premorbid functioning. Researchers tend to operationalize “recovery” for the purpose of studying prevalence rates and change after interventions. These studies use different approaches to defining recovery including whether people continue to meet CFS/ME diagnostic criteria, employment status, fatigue/functioning scores compared with population norms or a healthy control group, self-ratings of improvement, and whether patients subjectively believe they are recovered, or a combination of some of these indicators (e.g., Deale et al., 2001; Knoop et al., 2007; Vercoulen et al., 1996; White et al., 2013). Nevertheless, views of patients and professionals on recovery may differ—physicians may consider a patient recovered when the patient does not consider themselves so, and vice versa (Young, 1982). Young (1982) suggests that understanding the difference between “healing” and “curing” is an important distinction. While curing refers to the process affecting pathological organic states or disease, healing refers to the process affecting “illness” (a person’s perception and experiences of socially disvalued states which may include, but is not limited to, biological disease state). These concepts allow for the occurrence of a cured body but not a healed patient, or conversely for healing to occur even when the body is not cured. As such, it is important that patient views on recovery from CFS/ME are documented.

It is still unclear what “recovery” means to people with CFS/ME. Harland and colleagues (2019) found that among children with CFS/ME and their parents, signs of recovery were relatively easy to define, but defining “complete” recovery was complicated and varied considerably between individuals. Using the concept of liminality—falling in between socially/medically constructed categories—Brown et al. (2017) explored the narratives of 16 participants reporting recovery from CFS/ME, who were recruited from various sources—mainly self-help groups. No clear point of transition from the liminal state of illness with CFS/ME to wellness was found; even participants who self-reported as “recovered” experienced this state as markedly different from the pre-illness one as they still needed to manage the demands they made on themselves, including resting and pacing. Here “recovered” participants described having one foot in the ill world and one foot in the well world, but reported prioritizing resuming a normal work and social life within this state (Brown et al., 2017). Other studies which touch on recovery report narratives that include increased appreciation of life and improved self-understanding (Parslow et al., 2017; Whitehead, 2006).

As in other health areas, research is likely to elucidate the deeper meanings, signposts, and tasks involved in recovery, as well as highlight less recognized positions on recovery (e.g., Faircloth et al., 2004; Hopper, 2007). While some patients are positive about “recovery” discourses, others may be more skeptical about the discourse, for example, given concerns that patients may feel pressured to recover, that there are different interpretations of the concept between patients and professionals, and that only some kinds of recovery story can be told (Coreil et al., 2004; Gordon, 2013; Woods et al., 2019). It is also important to understand how sociocultural influences might impact on illness experience and recovery meanings. For example, HIV was historically constructed as a gay epidemic, but this sociocultural perception may be experienced as marginalizing for heterosexual people, who now turn up for HIV care in similar numbers to men who have sex with men in the United Kingdom (Persson, 2012). In terms of recovery, sociocultural influences may place particular expectations on how an illness should be managed, or it may be that the patient is not expected to recover, or that they are expected to recover in a short time frame. To better understand what it is like to live with a constraining illness such as CFS/ME, and how such lived experience influences perceptions of recovery, we use Talcott Parsons’s theory of the sick role (Parsons, 1951) as a lens with which to explore patient perceptions of recovery from CFS/ME.

### Theory: The Sick Role

In the early 1950s, prominent U.S. sociologist Talcott Parsons introduced a theory of social systems (Parsons, 1951), which included descriptions of different “roles” in society, with expectations/responsibilities placed on people in those roles, and societal judgments based on how people fulfill such roles. The “sick role” was one such role, which according to Parsons’s theory was entered into with a physician’s diagnosis. Entering this role was thought to free a person from some social expectations (e.g., work) and blame for being sick, while they temporarily occupied the role (Parsons, 1951). However, the role is considered an irregular or dysfunctional societal role and is governed by a range of expectations and responsibilities, including the “sick” person wanting to get well and “submitting” to medical experts to do so.

Subsequent directions in health and illness challenged a number of aspects of the sick role, and there are extensive critiques of the Parsonian construct (e.g., Rier, 2000). For example, neoliberalism emphasizes individual responsibility when managing and maintaining one’s health (Vassilev et al., 2017). Rather than submitting to medical experts, patients are increasingly expected to self-manage their condition to minimize (costly) health professional contact (Hallowell et al., 2015). In addition, rather than the sick role being bestowed (or not) by physicians, individuals are seen as having an active role in narrating (i.e. constructing) their illness experience (Frank, 2016), as well as in shared decision-making (Knight et al., 2018). Nevertheless, some researchers have argued that dismissing the sick role theory outright risks overlooking critical aspects which may be relevant today (Varul, 2010; Williams, 2005). While acknowledging its limitations, research has demonstrated parts of the theory’s usefulness in understanding current illness experiences. For example, Hallowell et al. (2015) found that the negative effects from elective risk-reducing breast surgery were either emphasized or minimized by women (i.e., sick role embraced or rejected), and that this was related to the amount of external legitimization they had received from health care professionals regarding undertaking surgery. They concluded that women were positioning themselves as co-creators of health and illness by either actively adopting or rejecting the sick role.

The applicability of the theory to chronic illness is more complex. Some suggest that the theory is based on the presumed temporary nature of illness, thus a poor fit for chronic health conditions (Segall, 1976). For example, those with chronic health conditions are less likely to be exempt from social obligations (e.g., employment), a defining characteristic of the sick role. In addition, there is likely to be more independence from health professionals, as those with a long-term condition take on more responsibility for managing their own illness, as reflected in the rise of patient groups among various types of longer-term conditions (Radley, 1994). However, others, including Parsons himself, argue that the theory highlights responsibility of the chronically ill person to minimize the effects of their health condition (rather than recover from it), by engaging with medical advice/treatment, displaying motivation to recover, and not “give in” to the illness (Varul, 2010). Bury (1982) proposes that a chronically ill individual may only have periods where they occupy the sick role due to, for example, symptom flare-ups or surgery. While the arguments go back and forth (Crossley, 1998), Varul (2010) argues that for the chronically ill there is a strong incentive (from a personal and societal perspective) for “normalization”—a return to societal roles despite persisting illness, with links to the current neoliberal imperative that one should be self-reliant when it comes to health (McGuigan, 2014). Given the apparent application of the sick role to chronic conditions, the current study used the Parsonian concept of the sick role to explore and interpret narratives around what recovery means to patients with CFS/ME. In particular, we explored how attempts to live with—and recovery from—illness shapes participants’ narratives around recovery.

## Method

### Design

This article reports on a qualitative secondary analysis (Heaton, 2000). The primary study investigated participants’ experiences of guided graded exercise self-help (GES) and changes in health and well-being (Cheshire et al., 2020). The secondary analysis, reported here, draws specifically on participants’ accounts and perceptions of recovery from CFS/ME within interview narratives. Ethical approval was obtained from the National (UK) Research Ethic Service Committee West Midlands—The Black Country on January 9, 2015 (reference 15/WM/0007). Written informed consent was obtained from all individual participants included in this study.

### Participants

Participants were taking part in a randomized controlled trial (RCT) *The Graded Exercise Therapy guided Self-hElp Trial* (*GETSET*; L. V. Clark et al., 2017) when they participated in the interview regarding their experiences of GES and living with/recovering from CFS/ME (Cheshire et al., 2020). Participants were recruited to the GETSET RCT (*n* = 211) at the time of being placed on the waiting list for treatment at one of two National Health Service (NHS) specialist CFS/ME secondary care clinics in the South of England (L. V. Clark et al., 2016). Study inclusion criteria included a diagnosis of NICE-defined CFS/ME (NICE, 2007). Patients were excluded if they could not speak and/or read English adequately, had current suicidal thoughts, had read the GES guide previously, received graded exercise therapy previously at one of the trial clinics, had physical contraindications to exercise, or were below 18 years old (L. V. Clark et al., 2017). For the interviews we invited only patients who had participated in the active arm of the GETSET RCT and had completed their follow-up questionnaire 12 weeks after randomization. We used the self-rated Clinical Global Impression (CGI) change scale (Guy, 1976) to identify suitable patients. The CGI is a brief validated measure that assesses subjective global functioning. Participants were asked to rate how much they thought their CFS/ME had changed since the start of the study on a 7-point scale, ranging from 1 “*very much better*” to 7 “*very much worse*.” Those who reported their CFS/ME as being improved (“much better” or “very much better”) or a “little worse” (no participant rated themselves as “much worse” or “very much worse”) were approached for the study, focuing on participants in varying stages of recovery. Participants meeting the inclusion criteria were posted an invitation pack including an invitation letter, patient information sheet, consent form, and prepaid envelope to return the consent form. The intention was to recruit 10 participants to each group (improved and deteriorated). Thirty-two potential participants were identified (14 “much better” [none rated themselves as “very much better”]; 18 “a little worse”) and were invited to be interviewed according to how recently they had finished GES. Our quotas were filled after we had invited 27 patients. Of these, eight declined. Therefore, nine participants who reported feeling “much better” and 10 who reported feeling “a little worse” provided consent and were interviewed.

Participants were predominantly female (*n* = 17) and of Caucasian ethnicity (*n* = 17). Mean age and interquartile range were 43 years (28–66) for the “a little worse” group and 39 years (21–54) for the “much better” group. The median length of time since onset of CFS/ME symptoms and interquartile range were 13 years (8–21) for the “a little worse” group and 4 years (3–5) for the “much better” group (Cheshire et al., 2020).

### Interviews

Interviews were arranged at a time and place most convenient for the participant. Eleven opted to be interviewed by telephone and eight face-to-face. Interviews used a semi-structured approach (Kvale, 2008). The interview topics included before and after intervention well-being, experiences of GES and perceptions of recovery from CFS/ME (see online Supplemental Material for interview questions). Interviews lasted between 13 and 80 minutes (*M* = 45 minutes). All interviews were audio-recorded with permission from the participant, transcribed verbatim, and returned to the participant for checking after anonymization.

### Data Analysis

Data were analyzed using thematic analysis (Braun & Clarke, 2006); the qualitative software tool NVivo (QSR International, 2011) was used to explore the data. Data reports relating to patients’ perceptions and accounts of recovery were generated in NVivo for both the deteriorated and improved groups (some patients who reported a deterioration by 12 weeks considered themselves improved at later interview). Initially adopting an inductive approach to the analysis, one researcher immersed herself in the data by repeatedly reading and annotating the reports. Key words and phrases were highlighted and underlined, and themes and ideas were written in the margins. Key findings were then listed in a separate document and refined to produce a draft list of themes. In the analysis, we used Parsons’s sick role theory (Parsons, 1951) as a sensitizing device, through which to interpret the themes. The application of sick role theory to the data was discussed and debated with the second author. Finally, themes were shaped around Parsonian theory and written up into the findings section.

## Results

Our analysis found that the meaning of recovery differed between participants, with expectations for improvement, the notion of the sick role, and associated stigma seemingly key influences on individual interpretations of their circumstances. These interpretations are explored through the following themes: Recovery: to return to, or regain; Leaving the sick role behind; Making recovery obtainable: Moving the goalposts; Rejecting the sick role: Critiquing recovery.

### Recovery: To Return to, or Regain

The dictionary definition of recovery is typically described as a “restoration” or a “regaining of” something (“Oxford English Dictionary,” 2019). In medical terms, this could be considered a return of one’s health to that before illness. Some participants in this study appeared to define recovery in this way, for example, being “100%” symptom free, or able to do everything other healthy people could do. These participants included those who had already achieved, or felt that they were close to achieving, a full return to health.

*I mean I’m getting there, as I say I’m about 80% there now, but I will get to 100%.*

*That to me is recovered because I can do what any other person was doing.*

*I probably would have said, in fact I think I did say to people that I thought I was recovered. But I’m not sure that you can ever say that, because at the moment, I’ve not felt, my 100 percent . . . . I’m probably at about 85 percent or something.*

However, defining recovery is not as straightforward for most other participants. Here we use the Parsonian concept of the sick role as a lens with which to explore how our remaining participants narrated their understanding of recovery.

### Leaving the Sick Role Behind

Some participants considered themselves “recovered” from CFS/ME, despite still experiencing symptoms related to the condition. For these participants, their symptoms had improved to the extent that they had been able to resume paid employment and other typical activities of daily living. Here, recovery was constructed to include “normality”; this appeared to mean living their lives in a more typical manner, such as fulfilling societal responsibilities, less interference from symptoms, and undertaking tasks of everyday living. It also included good “levels of wellness,” an improved ability to listen to the body, as well as effective management of symptoms/lifestyle adaptations, thus not requiring medical advice. These participants’ accounts appear to be consistent with Parsonian theory in terms of being able to largely leave the sick role behind through resumption of societal roles rather than the freedom from symptoms.

*I wouldn’t say I’ve got the illness, I wouldn’t say I suffer with pain particularly at all really, that’s really gone. But yeah some of the symptoms I might experience sometimes, I kind of take them as a signal, and then I do something about it . . . . So for instance, not having much sleep last night, been talking about this now, I know that I’m gonna feel drained so I’ll go to bed early. Whereas before all of this, I could kind of just, kind of keep, I might be a bit tired but I would just keep going kind of thing. But yeah not, not so much these days.*

*I still do my exercises and my stretches, I walk thirty minutes everyday. My alcohol, I’m obviously not drinking at the moment but I kept my alcohol to a minimum . . . there used to be a Head and Shoulders advert and they’d say someone would see it in the bathroom shelf “oh head and shoulders, I didn’t know you had dandruff” and the person would say “I don’t.” I feel like that about ME. Like oh you do all these things because you have ME. Well I do all these things because without them then I probably would [have ME], but they mean that I’m ok.*

For one participant, the importance of leaving the sick role behind was reinforced by her perceptions of the stigma associated with CFS/ME, in particular that it did not represent a real illness. This delegitimization of the illness created further challenges for the participant in terms of adopting the sick role.

*You said to me do you consider yourself to have ME and my initial reaction was like, oh God no I’m fine and in reality, I obviously am managing it but maybe I, I hate the label because of the stigma. So for me it’s like I manage the symptoms that my body presents me with but I can’t bear that bloody label because it’s got such a bad stigma. I had a school friend who had it when she was a teenager . . . and my parents . . . just went on about what a fake illness it was.*

### Making Recovery Obtainable: Moving the Goalposts

Many participants in this study viewed full recovery as something that they “hoped” to achieve, but thought ultimately it was unlikely to be obtainable. These beliefs were underpinned by a range of factors including the length and severity of their illness (many had been ill for more than 10 years), their advancing age, comorbid conditions, comments from health professionals who told them they would never fully recover, lack of treatment available for CFS/ME, and unsuccessful treatment experiences in the past.

*I think because I’ve had it for so long . . . I feel like I’ll probably be this way for the rest of my life. I can’t imagine being any different. As much as I’d love to and I’d want to, I would try anything to make it better but I can’t really see light at the end of the tunnel.*

*By the time I went to the hospital last year I’d had it 9 years, and I’d mostly no help except from reading for myself in the first 6. And I think if I’d had better help and therapy then, and maybe the therapy would’ve helped then.*

While some of these participants with little hope found describing their recovery challenging, others appeared to respond by moving their “recovery goal posts”—highlighting the achievement of obtainable goals over a full return to health. This was often described in terms of negotiating levels of normality, defined in terms of regaining functionality (e.g., ability to carry out everyday tasks, part-time work), having a “normal” reaction to physical activity (e.g., a more typical recovery time after exertion), experiencing “normal” versus “ME tired” (e.g., fatigue related to exertion that goes away after rest), or not waking up in pain. Others came to consider their life before CFS/ME an undesirable state, which had contributed to their becoming ill in the first place, a place they did not want or need to return to, “I don’t feel I have to recover enough to go back to that sort of lifestyle.” Negotiating this new “normality” seemed to allow participants some kind of relief in terms of the sick role, allowing people to respond more constructively to the needs of society, rather than just the needs of their body.

*I think what it [recovery] would mean being able to do normal things, like going to the shops without needing to sit down every 10 minutes, and not being totally wiped out after an hour’s shopping. Being able to go to the supermarket, which I don’t do because my husband does it, like he does it because I can’t cope with it.*

*There’s two different tiredness. People don’t get it! Like even when I was like “oh I’m so tired” everyone’s like “so am I, I’ve just been at work all day” and I’m like, yeah but you don’t understand it’s totally different, totally different. So they can go to bed that night and get up in the morning and feel refreshed and start the day again.*

Ideas about recovery can also become less related to achieving an unobtainable “normality” and thus leaving the sick role, and more focused around the pursuit of personally rewarding and meaningful activities, such as creative endeavours and being able to have fun.

*Most of the time it’s very dull, whereas if, during the good times if I can do even just something nice and creative or fun, then suddenly it makes such an enormous difference to your life. So, even if I don’t recover completely but if I got to a stage where I could do some fun things, maybe have a bit of social life, occasionally go, not be housebound anymore, travel home to [country where she’s from] to see my family because I’m not able to do that at the moment. I would be very grateful for even very small things, but to be honest, full recovery seems so, so far away at the moment that I don’t really, it’s almost like too good to be true so I don’t quite dare to even hope for that sort of thing.*

### Rejecting the Sick Role: Critiquing Recovery

There can be more disruptive and critical approaches to recovery, and not everyone considers it a useful construct. Two study participants challenged the idea of recovery as a goal to be obtained. One participant went on to describe how she rejected the idea that there was a specific role in society that “should” be fulfilled and explained how notions of recovery could result in the belief of the existence of an end point, with attempts to reach this goal detracting from living in the present moment in life.

*That word, recovery, . . . I don’t see recovery as something that is, I hate the analogy of the light at the end of tunnel. I don’t think life works like that. Realistically there’s never going to be something that is a beginning and end. That’s not how it works . . . . All of the people that I know of at the private clinic have all gone through a process of having to learn to understand what their body is doing, becoming in tune with that and then finding ways to slowly but surely improve. And some of them have reached a level where they’re capable of living at a perfectly healthy average, normal . . . It isn’t going to happen if you’re focused solely, or one is focused solely on the end game. I don’t know that in that instance a person is really focused on what’s happening in the time that’s it happening. It takes away their focus, a person’s focus. If it’s like, oh I’m going to get there eventually, I’m OK, I personally don’t believe that exists. But that’s a good thing for me because I think it’s made me very much live in the now.*

## Discussion

In this study we aimed to examine narratives of living with—and recovering from—CFS/ME, to shed light on what recovery means to patients with CFS/ME. The meaning of recovery differed between participants, with expectations for improvement, the Parsonian notion of the sick role, and associated stigma being key influences on individuals’ interpretations of what recovery meant for them. Many participants deployed the concept of recovery, but definitions varied; such wide and highly individualized definitions have also been found among children with CFS/ME and their parents (Harland et al., 2019). For the majority, definitions focused around functional improvement and ability to undertake life roles again, such as working. However, other definitions ranged from total freedom from symptoms, to being able to pursue personally rewarding and meaningful activities. Others redefined recovery as an altered lifestyle, taking into account those factors that led to their becoming ill in the first place. Yet other participants found notions of recovery to be less helpful to their circumstances, with some rejecting the concept outright. Indeed, science philosopher Canguilhem believed that a return to the pre-illness state was not possible because the experience of being ill inevitably alters the person, even their memory of being unwell will have some sort of impact (Canguilhem, 2012). In this section, we explore these findings in more detail and discuss their implications for clinicians and researchers working in the area.

Expectations about improvement was a key influence on how recovery was understood by each individual. Participant beliefs in the possibility of their attaining recovery were influenced by a range of factors, the most important being the length of time they had experienced their illness. Once participants had been ill for a significant amount of time (many had been ill for more than 10 years), the possibility of a full recovery (a return to pre-illness wellness) seemed more remote to participants. Such expectations are in line with the literature which suggests that the longer individuals have been ill when they receive treatment, the less successful it is likely to be (M. R. Clark et al., 1995; Vercoulen et al., 1996). Length of illness also combined with other factors to influence expectations. These factors included the severity of their illness, the presence of co-morbid conditions, feedback from some health professionals who told participants that they would never fully recover, the lack of treatment options for CFS/ME, and the accumulation of unsuccessful treatment experiences in the past. Thus, while participants may desire a full return to health, it may not be possible. For our participants, the more remote recovery seemed, the more that they then looked for other ways of re-framing their recovery, operationalizing the concept in ways that were more realistic and achievable to them. Other studies too have found that factors such as length of illness have important implications for patient experience and managing illness (e.g., Paterson et al., 1998). For our study, we found the Parsonian concept of the sick role useful in helping us interpret meanings participants assigned to recovery.

Parsons considered being sick an undesirable social role. Although the role excuses the sick from some societal responsibilities (e.g., work), there is the expectation that those occupying the role should seek to leave it as soon as possible (Parsons, 1951). While many criticisms have been leveled at the theory, our research found (as with other recent studies; Glenton, 2003; Hallowell et al., 2015) elements of the theory useful. Participants in our study (along with others; i.e., Harland et al., 2019) defined recovery not as being symptom free, but being able to resume certain societal roles, such as being in paid employment and/or being able to undertake typical activities of daily living. The desire to be “normal” was often used to express this state—echoing the deviancy which Parsons allocated to the sick role. Thus, our participants often defined recovery as consistent with leaving the sick role behind.

“Normalization” of life has been identified as a key coping strategy in long-term health conditions (e.g., Joachim & Acorn, 2000; Robinson, 1993), allowing a resumption of pre-illness roles to various degrees; how this manifests will differ between health condition and circumstances (Deatrick et al., 1999). It is widely acknowledged that the onset of chronic illness challenges an individual’s sense of identity, as well as how they perceive themselves and their life, the effects of which can be substantial. In seminal work by Bury (1982), chronic illness was described as a “biographical disruption,” an event that interrupts an individual’s life, throwing into relief their cognitive, emotional, and material resources. Social relationships and the ability to mobilize resources are also unsettled. Similarly, Charmaz (1983) describes how this disruption interferes with an individual’s sense of identity and can manifest as a “loss of self.” Chronically ill individuals often attempt to “normalize” in the face of this loss/disruption by, for example, attempting to continue with their employment even if this is challenging. They resist departing from “normal” behavior due to the disadvantages this carries—although some suffering may be alleviated here if formal or informal relationships (such as work and friends) allow some degree of flexibility on this issue (Bury, 1982). Chronic illness challenges individuals to reconstruct their lost/disrupted identity. Narratives are a way to create and give meaning to social reality, and patients give meaning to their illness through personal stories, allowing for the reworking of their lives in a way that enables the reconstruction of their identity and new ways of coping (Hyden, 1997). Here our participants “normalization” narratives appear to create opportunities to construct more useful realities for themselves, which allow them to better resist occupation of the sick role.

The literature on CFS/ME documents the struggles of people to be taken seriously by health professionals and society (Pilkington et al., 2020). The lack of a diagnostic marker or test has left those with the illness open to not being believed or taken seriously (Chew-Graham et al., 2009; Raine et al., 2004). Wanting to leave behind the stigma of a CFS/ME diagnosis was cited by one participant as why she defined herself as recovered while not being symptom free (Joachim & Acorn, 2000). Stigma of the condition appears to increase the sense of deviancy associated with the sick role, thus increasing the desire to leave the role behind. Interestingly, Parsons considered the sick role to be bestowed upon an individual by a physician’s diagnosis. As all participants in this study had a physician’s diagnosis, there appears to be some challenge to the physician’s role in legitimization of the condition; this may relate to the chronicity of CFS/ME (Parsonian theory was originally described for acute conditions), alternatively a poor understanding of an illness within the medical community generally has been shown to have a delegitimizing effect on diagnosis (Boulton, 2018). Sociological literature describes how diagnosis is “infused with symbolism,” which can have a significant impact on the subsequent experiences of the diagnosed (Jutel, 2017). More specifically, interactions with others can mean those with CFS/ME (or other invisible illnesses) experience a delegitimization of their experiences, which can lead to isolation, increased suffering and feelings of shame (Kleinman, 1992; Ware, 1992). For CFS/ME, the most demoralizing delegitimization appears to involve perceptions or experiences of illness as disbelieved, trivialized, or dismissed as psychosomatic (Bayliss et al., 2014; Ware, 1992).

Wanting to escape the sick role may be problematic if the individual is not physically able to do so. A boom and bust cycle can occur in CFS/ME (i.e., individuals can do a lot when they feel well [boom], which then results in increased symptoms or a setback [bust]) (King et al., 2020). While there is some disagreement between the medical profession and patients/patient charities as to which management approach for CFS/ME is best (Mallet et al., 2016), all approaches (graded exercise therapy, cognitive behavioral therapy, pacing) agree that the boom and bust cycle is undesirable (Goudsmit et al., 2012; Van Houdenhove & Luyten, 2008). The key CFS/ME symptom, postexertional malaise, may be partially responsible for this cycle as it leaves individuals unsure as to how far they can push themselves (as symptoms may be delayed). However, this research suggests that a desire to leave the sick role behind may cause those with the condition to adopt unhelpful management approaches. This predicament for people with CFS/ME is reflected in Radley’s writings on the sick role, which highlight the continuous struggle for people living with chronic illness to negotiate between the demands of society and demands of the body (Radley, 1994). Indeed, Brown et al. (2017) found that those who consider themselves recovered from CFS/ME still need to continue to manage their health and symptoms.

Attempts to leave the sick role behind also set up challenges for non CFS/ME patients too (Radley, 1994). For instance, this predicament was demonstrated by Glenton (2003) who found that patients with chronic back pain were both expected to continue to fulfill societal expectations and to conform to sick role responsibilities of seeking a medical diagnosis to legitimize their condition, seeking medical advice, and engaging in treatment. This challenge was exacerbated for participants by an absence of objective illness signs, lack of treatment options available, and fluctuation in symptom severity—issues which are all relevant to those with CFS/ME. In addition, Crossley (1998) found that those living with HIV (and HIV-related illness) sought to distance themselves from the sick role society had assigned them by downplaying medical knowledge/authority on their illness, asserting experiential authority (the expert patient), and rejecting some of the obligations placed on them by the sick role (e.g., safe sex, right to have children). Thus, a challenge for the chronically ill is set up: how to “live with illness in a healthy world” (Radley, 1994).

Our findings suggest that the helping professions would be wise to consider the potential conflicts and struggles inherent in CFS/ME, which have important implications for effective management of the condition. Working therapeutically in this area involves exploring patients’ internalized stigmatization and societal messages around the condition. Professionals could usefully focus on key goals for patients which are likely to be regaining functional ability to undertake typical activities of daily living, being able to undertake fun/leisure activities and/or full recovery, depending on the patient. NICE CFS/ME guidelines (NICE, 2007) highlight the importance of an individualized approach to care. Our finding—that recovery means different things to different people—supports this approach. Some may find discussions of recovery unhelpful, either because they feel that it is unobtainable or because they reject the concept of recovery altogether or societal standards of what “normality” entails. This is consistent with approaches to recovery from other long-term conditions where the notion of recovery—or at least a return to the pre-illness self—is considered unhelpful or not possible (Canguilhem, 2012; Kendrick, 2008; Robinson, 2016; Woods et al., 2019). Narratives from our participants suggest that ideas from mindfulness (living in the present moment without judgment), the body positivity or neutrality movements (which advocate the acceptance of all bodies no matter what their appearance or functionality), and focusing on happiness or meaning in life (e.g., Seligman) could provide alternative frameworks to explore with patients. Research has also shown that acceptance and commitment therapy, which has a focus on the individual’s life values and goals, can be helpful for CFS/ME (Jonsjö et al., 2019).

Our study also has implications for how recovery is measured. Devendorf et al. (2019) found that physicians believed that patients should be the ones to define whether or not they are recovered. While including patients’ views on recovery would be an important improvement in practice, our findings suggest that we cannot assume all participants will have the same definition of recovery. Any outcomes related to participant-defined recovery would need to be interpreted with the complexity that self-definition raises, where patient interpretations of recovery are grounded in their own personal circumstances and beliefs.

## Limitations

There are strengths and limitations to our study. There are some limitations in the applicability of sick role theory in relation to our participants, specifically in relation to the temporal course of illness. Sick role theory was specifically developed in regard to acute conditions, assuming that illness is temporary. Having an illness long-term changes the fundamental experience of the illness; for example, individuals tend to be less dependent on health professionals, assume greater autonomy in the management of their condition, and/or make greater use of patient and lay support. Despite these limitations, the theory has proved useful in increasing our understanding of meanings of recovery among our sample. Participants included both those who had benefited from an intervention and those who had not, providing a range of views on recovery which allowed us to explore the significant complexity around the concept for this population. Nevertheless, as with qualitative research, our sample was relatively small (*n* = 19) and the severely affected were not recruited to our study. Therefore, there may be views and experiences not uncovered by our analysis. In addition, we asked participants “What does recovery mean to you?,” consequently we elicited very personal narratives around recovery. Had we asked, “How should recovery for people with CFS/ME be defined?” we may have got different answers. Future research may wish to explore these two positions on recovery for people with CFS/ME and how they might be similar to, or diverge from, one another.

## Supplemental Material

sj-pdf-1-qhr-10.1177_1049732320969395 – Supplemental material for Sick of the Sick Role: Narratives of What “Recovery” Means to People With CFS/ME Click here for additional data file. Supplemental material, sj-pdf-1-qhr-10.1177_1049732320969395 for Sick of the Sick Role: Narratives of What “Recovery” Means to People With CFS/ME by Anna Cheshire, Damien Ridge, Lucy Clark and Peter White in Qualitative Health Research
