# Supplementary material for: Sick of the Sick Role: Narratives of What “Recovery” Means to People With CFS/ME
Source: Qual Health Res. 2020 Nov 11;31(2):298–308. doi: 10.1177/1049732320969395 (PMC7750673; doi:10.1177/1049732320969395)
Supplement: sj-pdf-1-qhr-10.1177_1049732320969395 – Supplemental material for Sick of the Sick Role: Narratives of What “Recovery” Means to People With CFS/ME [file sj-pdf-1-qhr-10.1177_1049732320969395.pdf]

Sample Questions

These questions are indicative of topics asked. Although the researcher covers the same topics in interviews, they will vary the way questions are asked to improve data collection:

1. If you can think back to immediately before you started your GETSET* treatment, can you tell me how the CFS/ME was affecting you at the time? [Prompt: can they recall the circumstances they found out about GETSET* if they are having difficulty remembering?]
2. Why did you decide to take part in GETSET*?
3. What were you expecting from treatment in the GETSET* trial? [Prompt: How was your experience of the trial different to expectations?]
4. Did you have any concerns about doing exercise during GETSET*? [Prompt: If yes, can you tell me about these?]
5. How was graded exercise therapy (GET) explained to you, if at all? What is your understanding of how GET works now? [Prompt: understanding of theory behind it].
6. What do you understand by 'setting a baseline'? [Prompt: Did you have trouble reaching baseline?]
7. What do you understand by the term 'exercise'? [Prompt: How does exercise affect your health?]
8. What happened when you started GET? –
9. To what extent did you stick to your GETSET* treatment programme?
10. How well did GET work for you?
    a. [Prompts: Compared with the way things were before the GETSET* trial? How well have you felt since the trial finished?
    b. Were there parts of the approach that worked better or worse than others? Please explain?]
11. Why do you think GET [did not work/worked] for you?
12. Were there any barrier(s) to doing GET? [Prompt: If yes, can you tell me about these?]
13. Was there anything in particular that helped you to do the GET? [Prompt: did anything help/make it easier for you to do GET]
14. How has your attitude to exercise changed having been in the GETSET* trial (if at all)?
15. To what extent do you feel you can get better [recover] from this illness? [Prompt: What does recovery mean to you?]
16. Was there anything important going on in your life at the time of GETSET* [Prompt: Could anything else have affected your wellbeing/participation in GETSET?*]
17. Did you see the graded exercise therapy treatment as physical exercise, physical activity, or something else? [Prompt: understanding of labels].
18. Is there anything else you want to say, that you have not had a chance to say it? [Prompt: Learnt anything? Social and practical support?]

*GETSET is the name of the randomised controlled trial in which participants were taking part: ***the Graded Exercise Therapy guided SElf-help Trial***
